# Genome and tissue-specific transcriptomes of the large-polyp coral, *Fimbriaphyllia* (*Euphyllia*) *ancora*: a recipe for a coral polyp

**DOI:** 10.1038/s42003-024-06544-4

**Published:** 2024-07-24

**Authors:** Shinya Shikina, Yuki Yoshioka, Yi-Ling Chiu, Taiga Uchida, Emma Chen, Yin-Chu Cheng, Tzu-Chieh Lin, Yu-Ling Chu, Miyuki Kanda, Mayumi Kawamitsu, Manabu Fujie, Takeshi Takeuchi, Yuna Zayasu, Noriyuki Satoh, Chuya Shinzato

**Affiliations:** 1 Institute of Marine Environment and Ecology, National Taiwan Ocean University, Keelung, Taiwan; 2 Center of Excellence for the Oceans, National Taiwan Ocean University, Keelung, Taiwan; 3 Marine Genomics Unit, Okinawa Institute of Science and Technology Graduate University, Onna, Okinawa, 904-0495 Japan; 4 Atmosphere and Ocean Research Institute, The University of Tokyo, Chiba, Japan; 5 DNA Sequencing Center Section, Okinawa Institute of Science and Technology Graduate University, Onna, Okinawa, 904-0495 Japan

**Keywords:** Transcriptomics, Transcriptomics

## Abstract

Coral polyps are composed of four tissues; however, their characteristics are largely unexplored. Here we report biological characteristics of tentacles (*Te*), mesenterial filaments (*Me*), body wall (*Bo)*, and mouth with pharynx (*MP*), using comparative genomic, morpho-histological, and transcriptomic analyses of the large-polyp coral, *Fimbriaphyllia ancora*. A draft *F. ancora* genome assembly of 434 Mbp was created. Morpho-histological and transcriptomic characterization of the four tissues showed that they have distinct differences in structure, primary cellular composition, and transcriptional profiles. Tissue-specific, highly expressed genes (HEGs) of *Te* are related to biological defense, predation, and coral-algal symbiosis. *Me* expresses multiple digestive enzymes, whereas *Bo* expresses innate immunity and biomineralization-related molecules. Many receptors for neuropeptides and neurotransmitters are expressed in *MP*. This dataset and new insights into tissue functions will facilitate a deeper understanding of symbiotic biology, immunology, biomineralization, digestive biology, and neurobiology in corals.

## Abstract (web summary)

A draft genome and tissue-specific transcriptome assemblies of the large-polyp coral, *Fimbriaphyllia ancora* were established. This dataset and new insights into tissue functions will facilitate a deeper understanding of coral biology.

## Introduction

Scleractinian corals are the primary builders of coral reef ecosystems, which nurture a wide variety of marine organisms^1,2^. Coral reefs support tropical and sub-tropical fisheries and tourism^3^. Despite their ecological and economic importance, biological characteristics of corals are still largely unknown. A comprehensive understanding of coral biology will not only allow more accurate predictions of anthropogenic impacts on corals^4^. but may help to establish methods for preservation and propagation of extant corals.

Corals belong to the phylum Cnidaria, a group of animals possessing cnidocytes, also known as stinging cells^5^. Corals and other cnidarians, e.g., sea anemones, hydras, and jellyfish, are classically regarded as diploblastic animals^5^. However, data suggesting a triploblastic nature have been reported^6^. They have simple body structures, and tissues, organs, and organ systems characteristic of vertebrates are not present^7^. Yet, individual coral polyps are composed largely of several functionally defined tissues: tentacle, mouth with pharynx, mesenterial filament, and body wall^5^. Tentacles, located atop each polyp, are responsible for predation, attack, and defense. The mouth with the associated pharynx is located in the upper part of the polyp not only to engulf prey, but also to release excreta, gametes, and larvae. Mesenterial filaments are located in the body cavity and constitute the primary tissue responsible for digestion and defense^8^. The body wall separates the interior of a coral polyp from the environment. Although differences and functions can be inferred from tissue locations and behavioral observations^9,10^, molecular and cellular characteristics and detailed functions of those tissues remain largely unexplored. Fine characterization of each tissue comprising coral polyps should promote better understanding of biological characteristics of corals.

**Fig. 1:**
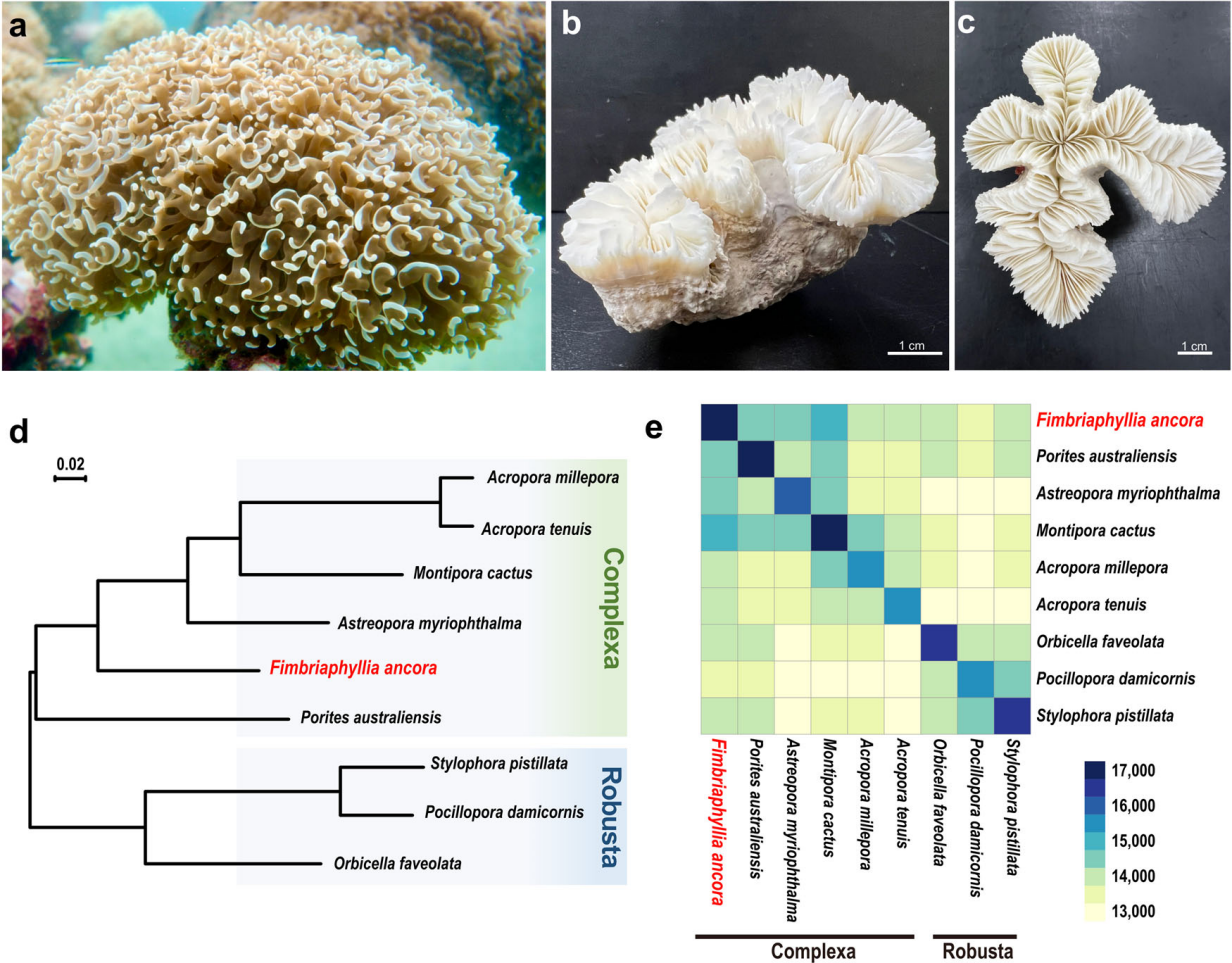
*Fimbriaphyllia ancora* and its phylogenetic relationships with other scleractinian corals. **a** External appearance of an *F. ancora* colony. **b**, **c** Top and side views of an *F. ancora* skeleton. The skeleton was photographed after removal of polyp tissue. Hammer or anchor-like tentacles and the flabello-meandroid skeleton typify *F. ancora*. **d** Molecular phylogeny of *F. ancora* based on 4208 single-copy Orthogroups (OGs) identified from published scleractinian genomes. All nodes are supported with 100% bootstrap values. **e** A heatmap showing numbers of shared OGs among scleractinian genomes.

Tissue-specific analysis is a powerful approach to characterize structure, cellular composition, and function. Detailed cellular and molecular analyses of isolated target tissues, rather than of whole individuals, allow us to explore differences in organization and function among tissues, as well as intrinsic mechanisms underlying specific functions^11,12^. However, tissue-specific analyses have rarely been conducted in corals. This is because the coral species often used for cellular and/or molecular studies, e.g., *Acropora millepora*^13^, *Pocillopora damicornis*^14^, *Stylophora pistillata*^15^, have very small polyps (about 1 mm in diameter), making tissue isolation difficult. *Fimbriaphyllia ancora* (family Euphyllidae), formerly called *Euphyllia ancora* (Fig. 1a), is distributed widely in reefs of the Indo-Pacific Ocean^16,17^, and is one of the most popular coral species in the aquarium industry^18^. Morphologically, it has tentacles with swollen, anchor-like tips and a flabello-meandroid skeleton (Fig. 1b, c)^16^. The most notable morphological characteristic is its polyp size (3–5 cm in diameter), and techniques for isolating tissues from these polyps have been established^19^.

Using *F. ancora*, we sought to determine cellular and molecular characteristics of the four major tissues (tentacle, mesenterial filament, body wall, and mouth with pharynx) constituting coral polyps. Ultimately, such fundamental data will enhance our understanding of biological characteristics of corals. To this end, we generated a draft genome of *F. ancora* and performed morpho-histological and transcriptomic analyses of all four polyp tissues.

## Results

### The *Fimbriaphyllia ancora* draft genome

In total, 14.6 Gbp of PacBio HiFi reads (QV > 20, 1.8 M reads, average length: 8145 bp) were obtained. Then, we assembled those reads into an *F. ancora* draft genome assembly of 434 Mbp, comprising 205 scaffold sequences with an N50 size of 5.18 Mbp (Table 1). The HiFi raw sequences had a single GC peak at around 40% (Supplementary Fig. S1a, b), and only a few reads (0.25%) mapped to reported Symbiodiniaceae genomes (BLASTN, <e^−10^). This indicated that there were very few contaminations of zooxanthellae sequences. A total of 27,537 protein-coding genes were predicted, and the number of genes was comparable to those of other coral genomes (Table 1^20–26^). Benchmarking Universal Single-Copy Orthologs (BUSCO) analyses^27,28^, which assess whether universal single-copy orthologous genes observed in more than 90% of metazoan species (from the OrthoDB database of orthologs) (www.orthodb.org; version 9) are recovered in a genome/transcriptome assembly, yielded completeness scores for the *F. ancora* genome assembly and gene models of about 95.6% and 96.1% (Complete BUSCO %), respectively. This indicated that the genome assembly and gene models are of comparable quality to those of previously reported coral genomes.

**Table 1:** Genome assembly and gene prediction statistics for *Fimbriaphyllia ancora* and comparisons with publicly available scleractinian coral genomes

Coral species	*Fimbriaphyllia ancora*	*Porites australiensis*	*Astreopora myriophthalma*	*Montipora cactus*	*Acropora tenuis*	*Acropora millepora*	*Stylophora pistillata*	*Orbicella faveolata*	*Pocillopora damicornis*
Total assembly size (Mbp)	434	576	373	640	403	475	400	486	234
Gap rate (%)	0.002	9.7	5.4	7.9	7.4	0.008	10.5	26.7	3.7
No. scaffolds	205	4983	1149	3532	1583	854 (14 chromosomes)	5687	1932	4393
Scaffold N50 (Kbp)	5180	555	1634	938	1166	19,840	457	1162	326
No. predicted protein-coding genes	27,537	30,603	25,406	29,158	22,905	30,136	24,833	25,916	19,922
BUSCO completeness % (upper: genome, lower: gene model)	C:95.6 [S:95.1, D:0.5], F:1.7, M:2.7 C:96.1 [S:95.2, D:0.9], F:1.9, M:2.0	C:91.6 [S:88.5, D:3.1], F:5.3, M:3.1 C:90.4 [S:86.8, D:3.6], F:5.9, M:3.8	C:93.4 [S:92.5, D:0.9], F:3.1, M:3.5 C:94.5 [S:93.2, D:1.3], F:3.1, M:2.4	C:92.1 [S:91.4, D:0.7], F:4.3, M:3.6 C:93.3 [S:92.5, D:0.8], F:4.1, M:2.6	C:91.9 [S:91.4, D:0.5], F:4.2, M:3.9 C:92.5 [S:91.7, D:0.8], F:2.9, M:4.2	C:92.5 [S:87.0, D:5.5], F:2.7, M:4.8 C:95 [S:88.6, D:6.4], F:2.0, M:3.0	C:92.5 [S:91.6, D:0.9], F:4.6, M:2.9 C:92.4 [S:91.9, D:0.5], F:4.6, M:3.0	C:85.1 [S:84.7, D:0.4], F:8.7, M:6.2 C:86.6 [S:83.6, D:3.0], F:7.1, M:6.3	C:90.7 [S:90.5, D:0.2], F:4.2, M:5.1 C:92.7 [S:92.6, D:0.1], F:3.4, M:3.9
Reference	This study	20	21	21	22	23	24	25	26

Next, orthologous relationships with other scleractinian corals were investigated using genome sequences of 4 acroporid species (*Acropora millepora*, *Acropora tenuis*, *Montipora cactus*, and *Astreopora myriophthalma*), *Porites australiensis*, *Stylophora pistillata*, *Pocillopora damicornis*, and *Orbicella faveolata*. We identified 23,701 orthologous gene families (OGs) from scleractinian genomes and obtained 17,128 OGs for *F. ancora*. Phylogenomic analysis of these anthozoan genomes using concatenated amino acid sequences of 4208 single-copy orthologous group genes (1,817,638 AAs) yielded robust phylogenetic relationships, with all clades supported by 100% bootstrap values (Fig. 1d), clearly indicating that *F. ancora* belongs to the Complexa coral clade, as reported in previous molecular phylogenic analyses (Fukami et al.^29^; Kitahara et al.^30^). Among the OGs in *F. ancora*, 10,016 are shared by all scleractinians, 16,511 OGs are shared with the Complexa group, and 371 gene families appear unique to *F. ancora* (Fig. 1e). The latter gene families comprise 1176 genes, of which 267 showed similarities to genes in the SwissProt database, whereas 277 resembled those in Pfam. These genes may have originated by gene duplication in the *Fimbriaphyllia* lineage.

### Morphological and histological characteristics of the four tissues comprising *F. ancora* polyps

**Fig. 2:**
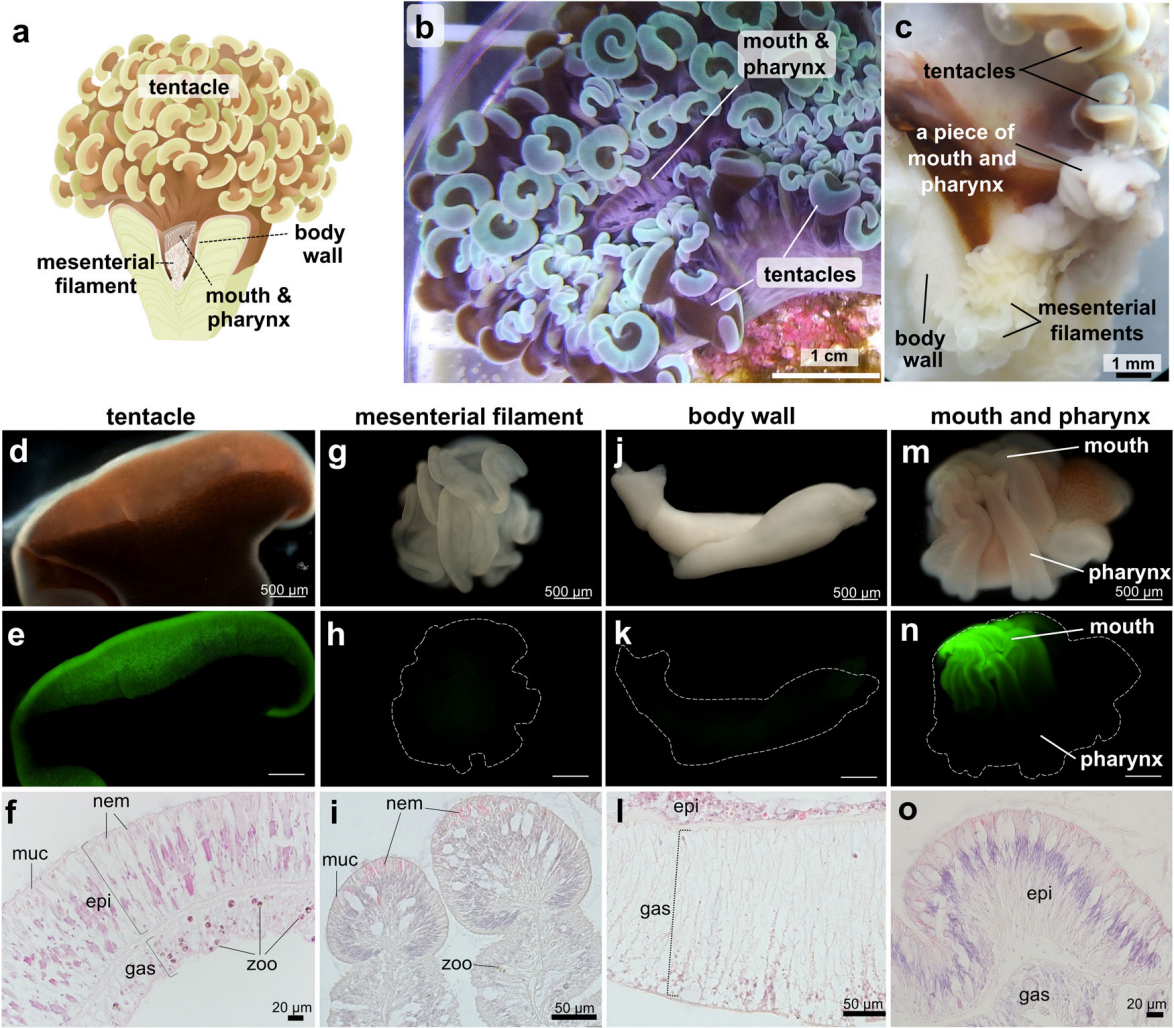
The tissues comprising *Fimbriaphyllia ancora* polyp. **a** A *F. ancora* polyp showing the 4 tissues analyzed in this study. **b** Top view of an *F. ancora* colony in an aquarium. The mouth is at the center of the polyp, and tentacles surround the mouth. **c** Representative picture of a dissected *F. ancora* polyp with tentacles, part of the mouth with the pharynx, mesenterial filaments, and part of the body wall. **d**–**o** Appearances of isolated polyp tissues and their histological micrographs. **d**–**f** A tentacle. **g**–**i** A mesenterial filament. **j**–**l** A piece of the body wall. **m**–**o** A piece of the mouth with the pharynx. **d**, **g**, j, **m** Bright views of isolated polyp tissues. **e**, **h**, **k**, **n** U‐MWIB 2 (GFP) filter view of the same field as in **d**, **g**, **j**, and **m**. Dashed lines in **h**, **k**, and **n** show outlines of each tissue. **f**, **i**, **l**, **o** Histological sections of isolated tentacle, mesenterial filament, body wall, and mouth and pharynx, respectively. epi epidermis, gas gastrodermis, zoo zooxanthellae, nem nematocyte, muc mucocytes.

Four major tissues: tentacle, mesenterial filament, body wall, and mouth with pharynx (Fig. 2a–c) were isolated, and their morpho-histological characteristics were examined (Table 2).

**Table 2:** External and histological features of each tissue comprising *F. ancora* polyps

	Appearance and other features	Green fluorescence	Tissue composition	Histological features
Tentacle	tubular shaped, white or light green at the tips, brown at the column, contains many zooxanthellae	strong expression	epidermis and gastrodermis	hollow structure, high dense of nematocytes are present in the epidermis, many zooxanthellae distributed in the gastrodermis
Mesenterial filament	undulating, white, contains few zooxanthellae	faint expression or undetectable	Pharyngeal epidermis gastrodermis	club shaped, nematocytes and mucocytes distributed at the margin
Body wall	membranous, whiter than other tissues, buoyant in seawater, contains few zooxanthellae	faint expression or undetectable	epidermis including calicodermis and gastrodermis	distinct two-layer structure, thin epidermis, porous but thick gastrodermis
Mouth and pharynx	radially furrowed, white or light gray, contains many zooxanthellae	localized expression	epidermis and gastrodermis	epidermis and mesoglea layers are thicker than other tissues, nematocytes and mucocytes distributed at the margin

#### Tentacle

Tentacles were basically tubular, and their tips were hammer- or anchor-like (Fig. 2a–c). They were hollow and expanded by retaining seawater inside. Each tentacle was 3–5 cm long and 1–2 cm wide when fully expanded. Tips were white to light green, and columns were brown, due to zooxanthellae (Fig. 2b). White areas at tentacle tips showed strong green fluorescence under a fluorescence microscope (Fig. 2d, e). Histologically, two distinct cell layers, epidermis, and gastrodermis, were observed. Epidermis was characterized by a high density of nematocytes and mucocytes, whereas gastrodermis cells and zooxanthellae were observed in the gastrodermis (Fig. 2f).

#### Mesenterial filament

Mesenterial filaments, located in the aboral part of the polyp, are formed by gastrodermal extensions toward the body cavity (Fig. 2a, c). Isolated mesenterial filaments were white to pale yellow with an undulating structure (Fig. 2g). Individual mesenterial filaments (when contracted) were 2–3 mm in diameter. Slight green fluorescence was observed (Fig. 2h). Histologically, the margins were distinctive and club-shaped, and only a few zooxanthellae were observed. A number of nematocytes and mucocytes were present in the marginal area (Fig. 2i).

#### Body wall

The isolated part of the body wall was white (Fig. 2j) and buoyant in seawater. Slight green fluorescence was observed under fluorescence microscopy (Fig. 2k). Histologically, the body wall consisted of a distinct two-cell layer, a thin epidermis containing a calicodermis layer, and a relatively thick, porous gastrodermis (Fig. 2l). Only a few nematocytes, mucocytes, and zooxanthellae were observed in the body wall.

#### Mouth with pharynx

Isolated pieces of mouth and pharynx were white to gray with a furrowed structure (Fig. 2m). Under the fluorescence microscope, spatial localization of green fluorescence was observed in the mouth, but not in the pharynx (Fig. 2n). Histologically, resembling tentacles, the mouth and pharynx consisted of two distinct layers, epidermis with many nematocytes and mucocytes and gastrodermis with many zooxanthellae (Fig. 2o).

### Transcriptome analysis of the four tissues

**Fig. 3:**
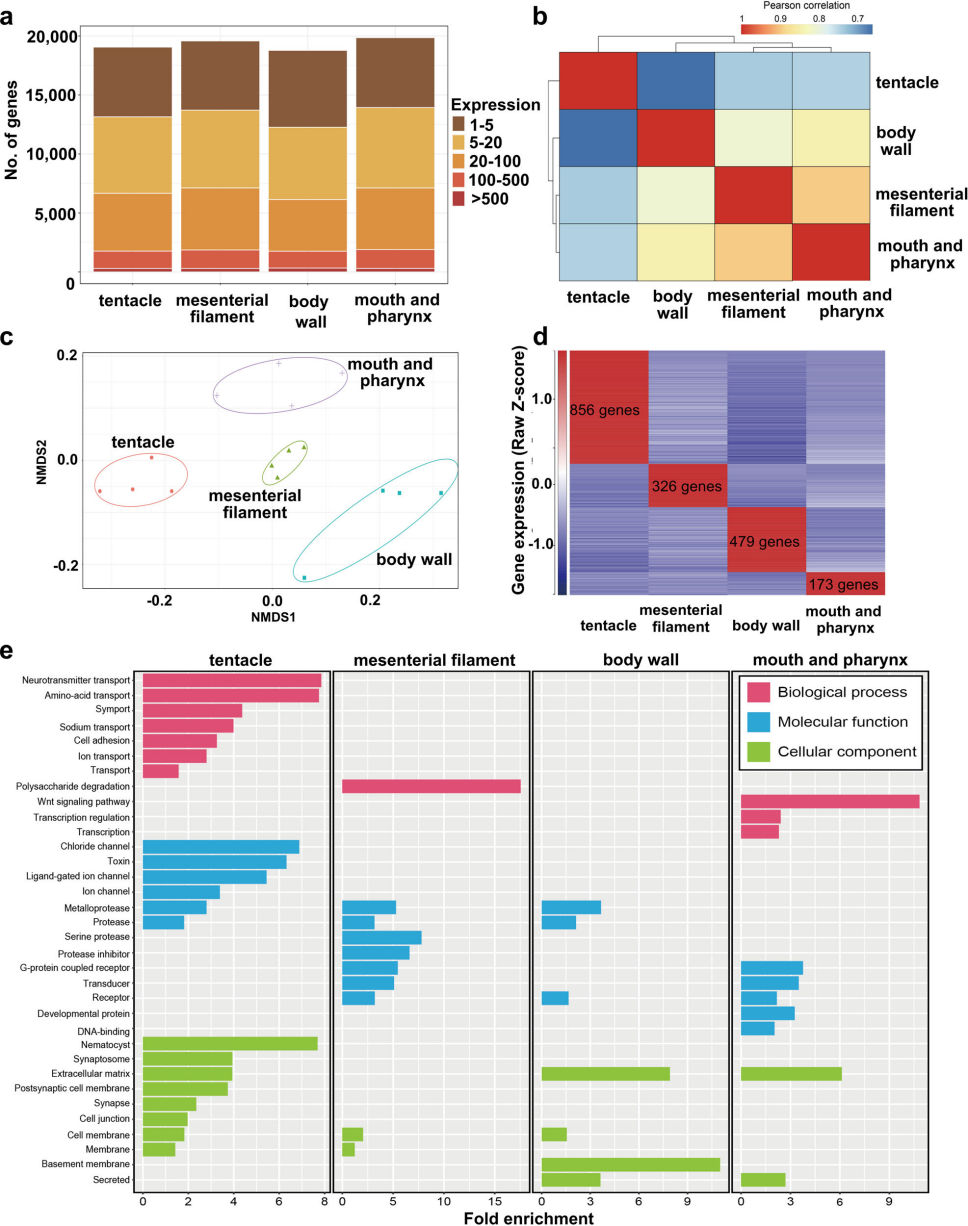
Classification of all protein-coding genes with regard to transcriptional levels in the four polyp tissues. **a** Total numbers of genes with detected transcripts in each tissue type using five abundance levels for transcripts per million (TPM) values; 1–5 TPM (brown), 5–20 TPM (dark yellow), 20–100 TPM (light orange), 100–500 TPM (dark orange), more than 500 TPM (dark red). In tentacle, 19,057 genes were detected, with 19,582 in mesenterial filaments, 18,777 in body wall, and 19,587 in mouth and pharynx. **b** Pearson correlation of tissue-specific transcriptomes of the four tissues using 16,703 expressed genes in all samples (TPM, >1). **c** Nonmetric multidimensional scaling (NMDS) plot based on Bray-Curtis distances of gene expression profiles from tentacle, mesenterial filament, body wall, and mouth and pharynx. 12,302 genes TPM > 1 in 16 samples) were used for comparisons. Colored lines surrounding each sample type represent covariance ellipsoids. **d** Relative gene expression levels of specifically highly expressed genes (total 1834 genes) in the four polyp tissues. TPM values were scaled to row Z-scores for each gene. **e** Significantly upregulated UniProt keywords detected from tissue-specific HEGs. Significantly (>4-fold change, *P* < 0.05) enriched UniProt keywords in biological processes (red bar), molecular function (blue bar), and cellular component (green bar). The X-axis represents the magnitude of fold enrichment. The Y-axis represents the functional category.

Numbers of genes expressed included 19,057 in tentacles, 19,582 in mesenterial filaments, 18,777 in the body wall, and 19,587 in the mouth with pharynx (Fig. 3a). Hierarchical clustering and a correlation heatmap showed that tentacles have the most specialized expression profiles (Fig. 3b). NMDS analysis further showed that transcriptional profiles of the four tissues were significantly different (Fig. 3c). In order to identify genes that characterize each tissue, we examined tissue-specific, highly expressed genes (HEGs): tentacles (856) mesenterial filaments (326), body wall (479), and mouth and pharynx (173) (*q*-value < 0.05, Fig. 3d). Functional enrichment analysis of HEGs (Fig. 3e) revealed tentacle-specific HEGs related to neurotransmitter transport, amino-acid transport, chloride channels, toxins, and nematocysts (>6-fold enrichment). Mesenterial filament-specific HEGs were related to polysaccharide degradation, serine proteases, and protease inhibitors, whereas body wall-specific HEGs were associated with basement membrane and extracellular matrix. In mouth and pharynx, genes associated with the Wnt signaling pathway were significantly enriched (Fig. 3e).

### Biological characteristics of the four tissues revealed by transcriptomic analysis

Based on results of morpho-histological observation and functional enrichment analysis of HEGs described above, we explored and further selected expressed genes characterizing each tissue.

#### Tentacles

**Fig. 4:**
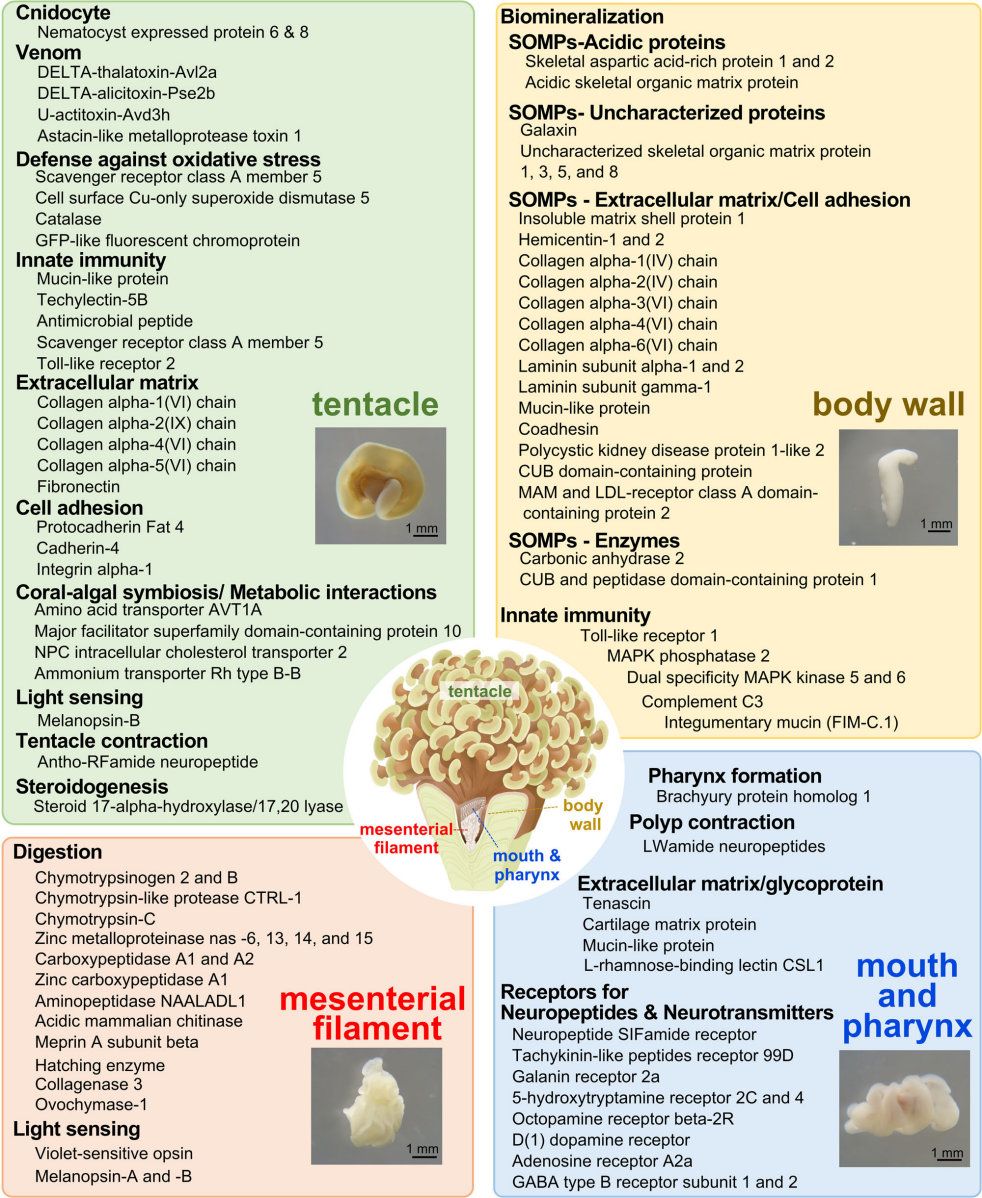
Possible functions of the four major tissues comprising *F. ancora* polyps and their potentially associated proteins. The micrograph in each panel shows isolated tentacle (left top), mesenterial filament (left bottom), body wall (right top), and mouth and pharynx (right bottom).

Tentacles highly expressed genes associated with biological defense and predation (Fig. 4). This included cnidocytes (*Nematocyst expressed proteins*), venom (*DELTA-thalatoxin, DELTA-alicitoxin, and U-actitoxin*), mucus (*Mucin-like protein*), innate immunity (*Techylectin-5B*, *Antimicrobial peptide*, *Toll-like receptor 2*, and *Scavenger receptor*), and defense against oxidative stress (*Cell surface Cu-only superoxide*, *Glutathione peroxidase 5*, *Catalase*, *GFP-like fluorescent chromoprotein*). Tentacles also highly expressed genes encoding extracellular matrix (*Collagen alpha-1,2,4, and 5*, *Fibronectin*) and a cell adhesion molecule (*Protocadherin Fat 4*), which may support the three-dimensional structure of tentacles. Genes associated with coral-algal symbiosis/metabolic interactions (*Amino acid transporter AVT1A*, *Major facilitator superfamily domain-containing protein 10*, *NPC intracellular cholesterol transporter 2 homolog*, and *Ammonium transporter Rh type B-B*) were also detected. Moreover, genes for the light-sensing molecule, melanopsin-B, and the precursor of Antho-RF amide neuropeptide, which is involved in tentacle contraction, were also highly expressed. Two genes encoding a steroidogenesis-related enzyme, Steroid 17-alpha-hydroxylase/17,20 lyase, were also highly expressed (Fig. 4).

#### Mesenterial filaments

Mesenterial filaments highly expressed a number of genes encoding proteases such as chymotrypsinogens, chymotrypsins, zinc metalloproteinases, carbopeptidases, aminopeptidase, meprin A subunit beta, and acidic mammalian chitinase. Mesenterial filaments also highly expressed genes encoding light-sensing molecules such as violet-sensitive opsin, melanopsin A, and melanopsin B (Fig. 4).

#### Body wall

Body wall highly expressed a number of genes encoding skeletal organic matrix proteins (SOMPs), e.g., acidic SOMPs, an uncharacterized protein, extracellular matrix, cell adhesion molecules, and enzymes (Fig. 4). Genes encoding molecules related to innate immunity (Toll-like receptor 1, MAPK 2/5/6, Complement C3, integumentary mucin) were also highly expressed (Fig. 4).

#### Mouth and pharynx

In mouth and pharynx, genes encoding a transcription factor, Brachyury, essential for pharynx formation during embryogenesis in corals, were highly expressed. Molecules related to the Wnt signaling pathway, innate immunity, and the precursor of GLWamide neuropeptides, involved in polyp contraction, were also detected (Fig. 4). Mouth and pharynx also highly expressed a number of receptors for neuropeptides, e.g., neuropeptide SIFamide, tachykinin-like peptides, and neurotransmitters, e.g., 5-hydroxytryptamine, octopamine, dopamine, GABA (Fig. 4).

### Tissue-specific expression patterns of specific gene families

**Fig. 5:**
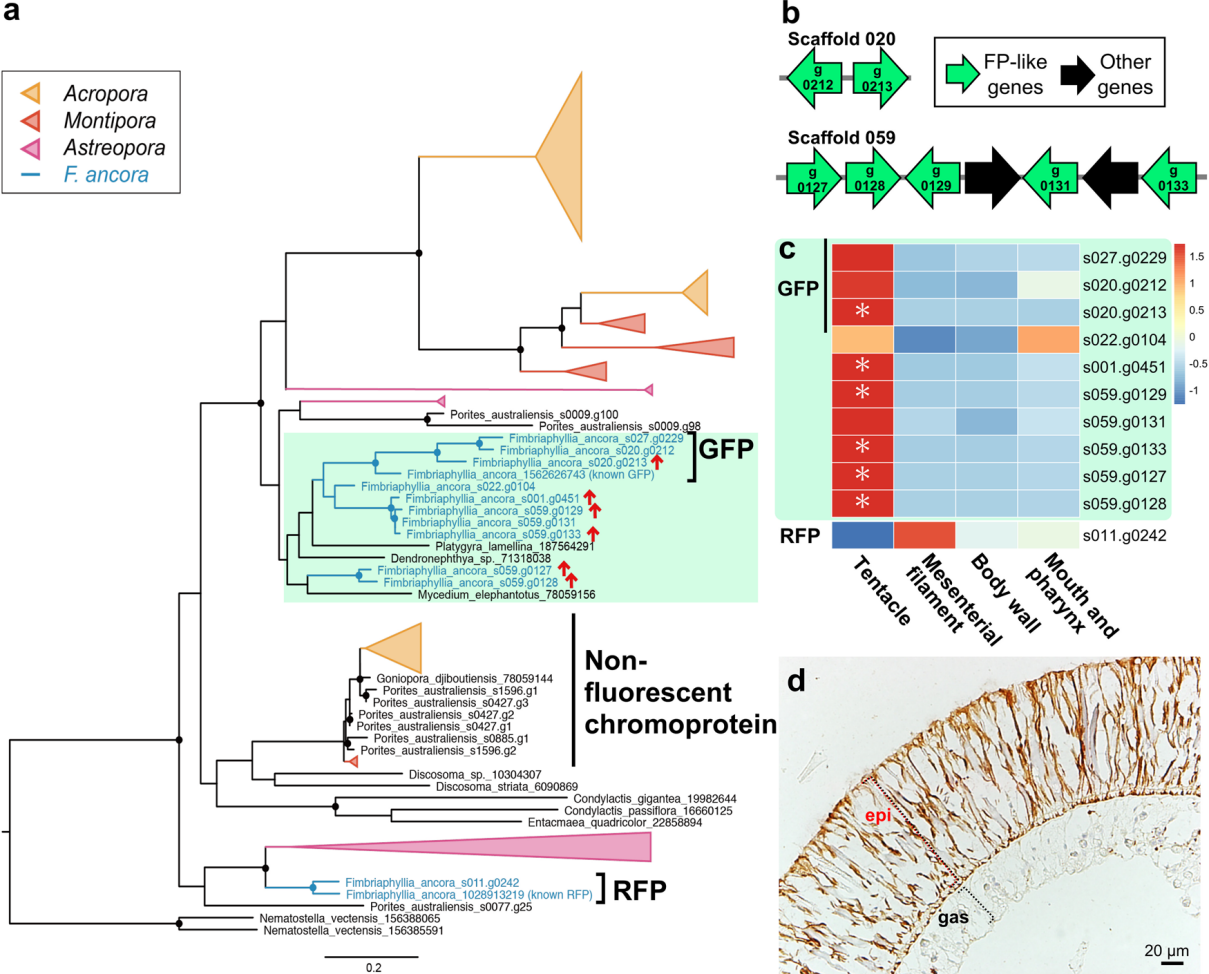
Tissue-specific expression patterns of fluorescent protein (FP) genes. **a** Molecular phylogenetic analysis of possible *F. ancora* FP-like genes with two known *F. ancora* FP genes (one GreenFP and one RedFP), FP genes, and non-fluorescent chromoprotein genes of diverse anthozoan taxa, including *Acropora*, *Montipora*, and *Astreopora* corals. Red arrows indicate tentacle-specific HEGs. Circles on branches indicate bootstrap values higher than 80%. **b** Arrangement and orientation of seven FP-like genes (shown as green arrows) in the *F. ancora* genome. Numbers in arrows indicate gene IDs in scaffolds. **c** A heatmap showing transcript levels of 11 FP-like genes in tentacle, mesenterial filament, body wall, and mouth and pharynx. Asterisks indicate tissue-specific HEGs. **d** Epidermal expression of GFP in tentacles as assessed by immunohistochemical analysis with anti-GFP antibody.

Coral color is primarily attributed to pigment proteins, including fluorescent proteins (FPs), non-fluorescent chromoproteins, and brown-pigmented zooxanthellae. In the *F. ancora* genome, we identified 11 candidate genes encoding FPs. Genes encoding non-fluorescent chromoproteins were not identified. Based on molecular phylogenetic analysis of two known *F. ancora* FP genes (one GreenFP and one RedFP)^31,32^ and FP-like genes from diverse anthozoan taxa, we estimated that 3 of the identified FP candidate genes encode GreenFPs (GFPs), while 1 encodes a RedFP (RFP) (Fig. 5a). Our molecular phylogenetic analysis also showed that 7 of 11 FP candidate genes in *F. ancora* were expanded by tandem duplication, independently of those in *Acropora* (Fig. 5a). These 7 FP genes are also arranged in tandem in the genome (Fig. 5b). Tissue-specific transcriptome analyses revealed that 6 of 11 FP candidate genes, including 1 GFP gene, were highly and significantly expressed in tentacles (Fig. 5c), in agreement with fluorescent microscopy. One FP-like gene, s020 g104, is also highly, but not significantly, expressed in the mouth and pharynx (Fig. 5c). Immunohistochemical analysis with anti-GFP antibody showed that GFP was expressed in epidermis of tentacles (Fig. 5d).

**Fig. 6:**
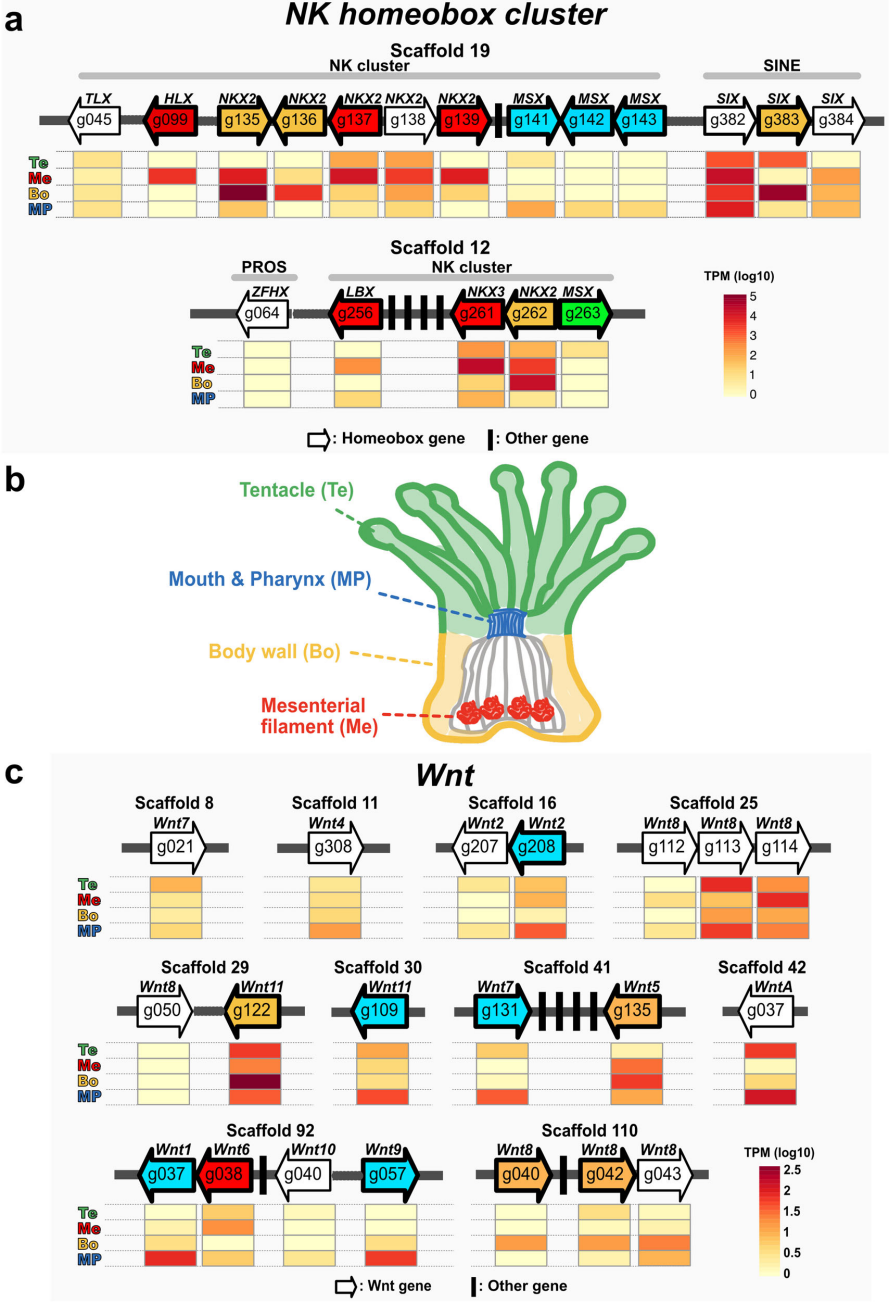
Tissue-specific expression patterns of NK homeobox and Wnt genes. **a** Organization of NK homeobox gene clusters in the *F. ancora* genome. Gene IDs and their relative positions and orientations in scaffolds 19 and 12 are indicated with arrows. Arrow directions indicate directions of transcription. Arrow colors indicate the tissue in which genes are highly expressed. Relative expression levels in the four polyp tissues are shown with heat maps under the arrows. Te tentacle (green), MP mouth and pharynx (blue), Bo body wall (yellow), Me mesenterial filament (red). **b** Schematic diagram of a coral polyp showing locations of the four tissues analyzed in this study. **c** Organization of Wnt genes in the *F. ancora* genome. Gene IDs and their relative positions and orientations in scaffolds are indicated by arrows. As in **a** above, colors of arrows indicate tissues in which genes are highly expressed. Relative expression levels in the four tissues are shown with heat maps under the arrows.

Homeobox genes function in developmental patterning in animals^33^. In the *F. ancora* genome, the Antennapedia (ANTP) class of homeobox genes, including Hox-like, ParaHox-like (Supplementary Fig. S2), and NK cluster genes, were identified. Tissue distribution analysis revealed that 8 of 10 NK genes cluster in scaffold 19 and all 4 in scaffold 12 showed tissue-specific expression patterns (Fig. 6a): 3 *MSX* genes in mouth and pharynx, two *NKX2* genes and one *NKX3* gene in body wall, *HLX, LBX, NKX3* and two *NKX2* genes in mesenterial filaments, and one *MSX* gene in tentacle (Fig. 6a, b).

The Wnt gene family encodes secreted signaling molecules that control cell fate in animal development and disease. In the *F. ancora* genome, 20 possible Wnt genes with conserved wnt family domains (PF00110) were identified (Fig. 6c, Supplementary Fig. S3). Tissue distribution analysis found that 10 of 20 Wnt genes were assigned as tissue-specific HEGs (Fig. 6c): 5 genes (*Wnt1,-2,-7,-9*, and *-11*) in mouth and pharynx, 4 genes (*Wnt5,-8,-8* and *-11*) in body wall, and 1 gene (*Wnt6*) in mesenterial filaments (Fig. 6c).

## Discussion

### Establishment of an F. ancora draft genome and the tissue-specific gene expression profiles of coral polyps

The present study established a draft genome and tissue-specific transcriptome assemblies of *F. ancora*. We previously created an *F. ancora* gonadal transcriptome assembly that allowed us to identify sex- and gonadal, phase-specific genes associated with germ cell development^34^. Altogether, tissue-specific transcriptomes of 6 tissues, i.e., tentacles, mesenterial filaments, body wall, mouth with pharynx, testes, and ovary, are now available for *F. ancora*. Although genomes of more than 25 coral species have been reported so far^4,20–26,35^, tissue-specific spatial expression patterns have not been undertaken in those species. These transcriptomic datasets of *F. ancora* not only shed light on unexplored functions of coral tissues, but provide a useful foundation for better understanding of gene functions.

### Tentacles: multifunctional tissues involved in biological defense, predation, external-factor sensing, symbiosis, and steroid synthesis

Our morpho-histological observations demonstrated that some nematocytes and mucocytes are located in the epidermis, whereas zooxanthellae are present in the gastrodermis of tentacles. Consistent with these observations, genes associated with defense and predation^36–39^, innate immunity^40–45^, and symbiosis (antioxidant defense, immune regulation, and metabolite exchange^46^ are highly expressed. Light-sensing molecule are also highly expressed^47,48^. These findings indicate that tentacles are multifunctional tissues that not only serve in biological defense, predation, and light-sensing, but are also central to symbiosis.

FPs create vivid displays of coral color^49,50^. To date, diverse functions of FPs have been shown. Since FPs absorb ultraviolet A and emit light of lower energy, the functions include photoprotection from high UVA/blue irradiation and photosynthetic enhancement of zooxanthellae^51,52^. In addition, FPs also serve antioxidant functions^32,53,54^, and participate in innate immunity^55,56^, stress response^57^, establishment of symbiosis with free-living dinoflagellates (family Symbiodiniaceae)^58^, and prey attraction^57^. Previously, we reported identification of RedFP (RFP) gene, specifically expressed in oocytes of *F. ancora*, and suggested its involvement in coral oogenesis^32^. The present study identified 6 FP genes that are highly upregulated in tentacles. The FPs may have acquired functions specific to tentacles such as protection of coral cells and zooxanthellae from intense UV light^51^, or in attracting free-living dinoflagellate ^58^and prey^59^.

Of particular interest is the significantly higher expression of the gene encoding steroid 17α-hydroxylase/17,20-lyase (Cyp17a), a key enzyme in production of sex steroids and cortisol^60^, in tentacles. Steroids are biologically active compounds derived from cyclopenta[a]phenanthrene^61^. They function as important components of cell membranes and are involved in a wide range of physiological processes, such as stress response, immune response, behavior, reproduction, etc^62^.. Steroid biosynthesis is catalyzed by activities of various steroidogenic enzymes^63^. To date, steroidogenic enzyme activities, plus estrogens and testosterone have been demonstrated in tissue extracts of some scleractinians^64–67^. Some genes encoding steroidogenic enzymes were also demonstrated^68,69^. Although further research is required to clarify transcript localization and steroidal activity, our findings imply that steroid biosynthesis occurs in tentacles, and that produced steroids/cortisol could be associated with some functions in tentacles, such as maintenance of cell membrane integrity, biological defense, or symbiosis.

### Digestion and light-sensing in mesenterial filaments

Our histological analysis demonstrated that many cnidocytes and mucocytes are present in the margins of mesenterial filaments. Further, we identified a variety of digestive enzymes that are highly expressed in mesenterial filaments. Enzymes similar to those expressed in digestive tissues of other cnidarians, such as the sea anemone, *Nematostella vectensis*, and the jellyfish, *Aurelia aurita*, were also identified^6^. Few studies have reported on the presence and expression of digestive enzymes in corals^70,71^. Therefore, this study may provide important insights into understanding the digestive system of corals. Since corals feed on various types of prey^72,73^, it is possible that expression levels of the identified digestive enzymes may also vary depending on prey.

Some coral behaviors such as tentacle expansion/contraction are strongly influenced by light^74–78^. Additionally, it usually releases gametes at night^76^. However, it is still largely unknown how corals, which have no special light-sensing organs, perceive light. In the jellyfish *Clytia hemisphaerica*, spawning timing is controlled by light, and that process involves the photosensing molecule, opsin, expressed in gonads^79^. In *Acropora millepora*, multiple opsins have been identified^47^, including one that responds to UV light^80^. In the *F. ancora* genome, there are 32 genes encoding opsins/melanopsins and some exhibit tissue-specific expression. Melanopsin B is highly expressed in tentacles and mesenterial filaments, whereas Violet-sensitive opsin, Melanopsin A, is highly expressed in mesenterial filaments. This suggests that corals may enhance sensitivity to light by highly expressing photo-sensing molecules not only in tentacles, but also in mesenterial filaments.

### Body wall: skeleton formation and a chemical-physical barrier

Nematocytes and zooxanthellae are nearly absent in the body wall of *F. ancora*, suggesting that body wall is little involved in predation or symbiosis. Surprisingly, body wall highly expresses a variety of molecules involved in biomineralization (acidic SOMPs, uncharacterized proteins, extracellular matrix, cell adhesion molecules, and enzymes)^81–83^. This is possibly because *F. ancora* forms its skeleton entirely outside the polyp body wall. The epidermis of the body wall basically comprises a layer of calicodermis, in contact with the skeleton. Further, the body wall also expresses some molecules involved in innate immunity, such as Toll-like receptor 1, Complement C3, integumentary mucin^40,44,84^. These results indicate that the body wall is not only the primary tissue for skeleton formation, but also functions as a chemical-physical barrier against parasitic and biofouling organisms.

### Mouth and pharynx: key tissues for the coral nervous system?

A notable finding was the high expression of many neuropeptides and biogenic amine receptors in the mouth and pharynx. Both peptidergic and non-peptidergic neurotransmission/neuromodulation are fundamental to cnidarian physiology^85–87^. In corals, GLWamide-positive and RFamide-positive neurons have been found in the mouth and pharynx, and are involved in polyp contraction^13,88,89^. Some neuroactive compounds such as dopamine induce larval settlement in the coral, *Leptastrea purpurea*^90^. Increases levels of neuroactive compounds were detected during a synchronous spawning event in *Acropora intermedia*^91^. Evidence for neuropeptides and neurotransmitters in coral physiology is slowly accumulating; however, many coral neuropeptides, neurotransmitters, and their receptors have not yet been identified in corals. Their future identification will lead to a better understanding of coral physiology, targeting especially the mouth and pharynx.

### Tissue-specific expression of NK homeobox clusters and Wnt genes

Homeobox genes were identified in the *F. ancora* genome, as reported in some other corals^92,93^. Notably, 11 of 14 genes in NK homeobox clusters showed tissue-specific expression patterns in *F. ancora*. In a sea anemone, *Nematostella vectensis* (Anthozoa), NK homeobox genes also show tissue-specific expression patterns. *Gbx* is expressed in pharyngeal endoderm^94^, *Hlx* and *Nk6* in pharyngeal ectoderm, and *Nk3* in nutrient-storing somatic gonads in mesentery^6^. Further detailed expression analysis in corals is likely to reveal other similarities and differences with *N. vectensis*.

Of particular interest is the discovery of significantly higher expression of genes encoding various Wnt proteins in *F. ancora* mouth and pharynx. In *Hydra*, various types of Wnt mRNA (*HyWnt1, -3, -7, -9/10a*, *-9/10c, -11*, and *-16*) are expressed in hypostomes of both adult polyps and new buds, and their involvement in head formation was demonstrated^95,96^. In *Nematostella*, induction of Wnt signaling with alsterpaullone results in formation of ectopic oral tissue during regeneration and embryogenesis^97^. Accordingly, Wnt may also be involved in formation of oral tissue in corals.

### Future perspectives

Given the serious plight of coral reefs, promoting coral conservation and increasing our understanding of coral biology is essential. Genomic information from *F. ancora* will facilitate coral conservation activities. Genome-wide SNP markers will reveal detailed population structures in nature^98–100^, and will be applicable to *F. ancora* aquaculture to monitor genetic diversity in captivity^101–104^.

Although morphohistological and transcriptome characteristics of coral polyp tissues are presented here, numbers of cell types and compositional ratios of cells constituting each tissue are still unknown. Single-cell transcriptome analysis^105^ of each coral tissue will clarify these issues in the future.

In conclusion, we have established a draft genome and tissue-specific transcriptome assemblies of *Fimbriaphyllia ancora*. The established dataset for each polyp tissue will not only shed light on unexplored functions of coral tissues, but provide a useful foundation for better understanding of gene functions.

## Materials and methods

### Sampling, DNA and RNA extraction, transcriptome and genome sequencing

Four colonies of *F. ancora* were collected on reef slopes in the nearshore of Onna Village, Okinawa, Japan, under Okinawa prefectural permit #30-8 in 2018. Four polyp tissues, tentacle, mouth, mesenterial filament, and body wall, were isolated and photographed under a stereomicroscope^19^. Collected tissues were snap-frozen in liquid nitrogen and stored at −80 °C until use. Total RNA was isolated from each tissue with an RNeasy Plant Kit (QIAGEN Inc., Valencia, CA). For transcriptome sequencing, a TruSeq Stranded mRNA Library Kit (Illumina, San Diego, CA) was used for mRNA sequencing and library preparation, and each library was sequenced from 150-bp paired-end reads using a HiSeq 4000 (Illumina). To prepare genomic DNA for sequencing, isolated mesenterial filaments from one colony were maintained in Petri dishes for 1 week with 50 mL of filtered seawater containing 0.2 mM menthol^106^ to eliminate symbiotic algae. Bleached mesenterial filaments were snap-frozen in liquid nitrogen and stored at −80 °C until use. Genomic DNA was isolated using the phenol-chloroform method, and sequenced on a PacBio platform to obtain highly accurate long-read sequencing data. Genome shotgun sequencing (150-bp paired-end) of the same DNA sample was also performed on an Illumina HiSeq4000.

### Genome assembly and gene prediction

PacBio HiFi reads with quality values > 20 were assembled with Hifiasm version 0.14-r312 using default settings^107^. Possible diploid scaffolds were first removed with Purge_haplotigs ver. 1.1.1^108^, and then merged with HaploMerger2^109^. Further scaffolding was performed with LINKS (ver. 1.8.7) with a kmer size of 21^110^. Possible errors in genome assembly were corrected with Hypo ver. 1.0.3^111^ using Illumina shotgun data with default settings. For gene prediction from assembled genome sequences, we used the above-prepared RNA-Seq data from the four tissues and RNA-Seq data from *F. ancora* male and female gonads in different developmental stages^34^. Low-quality reads (quality score <20 and length <20 bp) and sequence adapters in RNA-Seq data were trimmed using CUTADAPT v1.18^112^. Repetitive elements in scaffolds were identified de novo with RepeatScout v1.0.6^113^ and RepeatMasker v4.1.0 (http://www.repeatmasker.org). Repetitive elements were filtered out by length (>50 bp) and occurrence. Gene prediction was first performed with the BRAKER pipeline v2.1.2^114^, with AUGUSTUS v3.3.3. RNA-seq reads were aligned to genome sequences with HISAT v2.1.0^115,116^. Then, alignment information was used for BRAKER gene prediction with options “UTR=on”, “soft- masking”, and “AUGUSTUS_ab_initio.” To improve gene prediction, we further executed genome-guided transcriptome assembly using StringTie^114^ with option “-m 500.” Genome-based transcript structure was predicted with TransDecoder (https://github.com/TransDecoder/TransDecoder/wiki). During read alignment, we used soft-masked repeats for genome-guided transcriptome assembly and hard-masked repeats for BRAKER gene prediction. Finally, genes present in genome-guided assembly or ab initio prediction, but absent in predictions from the hint file were added to the predicted file using GffCompare^117^. We assessed completeness of the genome assembly and gene prediction with Benchmarking Universal Single-Copy Orthologs (BUSCO) ver. 5.2.2^27,28^ using the Metazoa set (978 genes).

### Gene annotation, clustering of orthologous genes, and molecular phylogenetic analysis

Predicted gene models were BLASTed against the Uniprot/Swissprot (UniProt Consortium 2018) database and were analyzed with InterProScan 5 with a cutoff of e^−5^^118^. To cluster orthologous genes in scleractinian genomes, we used four acroporid species (*A. millepora*, *A. tenuis*, *Montipora cactus*, and *Astreopora myriophthalma*^4,21–23,35^, *Porites australiensis*^20^, *Stylophora pistillata*^24^, *Pocillopora damicornis*^26^ and *Orbicella faveolata*^25^. For the *A. millepora*, *S. pistillata*, *O. faveolata*, and *P. damicornis* genomes, we downloaded data from the NCBI RefSeq database. Then, using OrthoFinder version 2.5.4^119^, we performed clustering of possible orthologs, and Orthogroups (OGs) were used for subsequent analyses.

For phylogenomic analysis of scleractinian genomes, we used 4208 genes that were identified by OrthoFinder as single-copy genes in all of the above scleractinian genomes. All amino acid sequences belonging to same OG were aligned with MAFFT (ver. 7.310. with –auto option)^120^ and all gaps in the alignment were removed with TrimAL^121^ with the –nogaps option. Then all sequences from the same species were concatenated. Finally, a maximum likelihood analysis was performed using concatenated sequences (1,817,638 amino acids in length) from RAxML (maximum likelihood method) with 100 bootstraps replicates and the “protgammaauto” option^122^. For phylogenetic analysis of each gene, amino acid sequences were aligned using MAFFT (ver. 7.310. with –auto option)^120^, and gaps in aligned sequences were trimmed using TrimAL^121^ with the –gappyout option. After that, poorly aligned sequences were removed (-resoverlap 0.75 -seqoverlap 80). Then we performed molecular phylogenetic analysis of the selected alignments using RAxML (maximum likelihood method) with 100 bootstrap replicates and “protgammaauto” option.

### Tissue-specific gene expression analysis

RNA-Seq data obtained from the 16 samples (4 tissue types × 4 colonies) were used. Low-quality reads (quality score <20 and length <20 bp) and Illumina sequence adapters were trimmed with CUTADAPT v1.16^112^, and then mapped to *F. ancora* gene models (mRNA) using SALMON v1.8.0. Mapping counts were normalized with the trimmed mean of M values (TMM) method, and then converted to counts per million (CPM) using EdgeR v3.32.1 in R v4.0.3. Gene expression levels (numbers of mapped reads) of each tissue were compared pairwise with the other three tissues. *p*-values were adjusted using the Benjamini–Hochberg method in EdgeR. When the gene expression level was significantly higher (False discovery rate < 0.05) in one tissue than the other three, genes were considered tissue-specific HEGs.

### Histology and immunohistochemistry

Histological and immunohistological analysis was performed according to methodology described previously^123^. Briefly, isolated tissues were fixed in filtered seawater containing 20% Zinc Formal Fixx (Thermo Scientific Shandon, Cheshire, UK) for 16 h and preserved in 70% ethanol until use. Dehydrated samples were embedded in paraplast plus (Sherwood Medical, St. Louis, MO), sliced into 4-mm serial sections, and stained with haematoxylin and eosin Y (H & E staining, Thermo Shandon). For immunohistochemical staining, hydrated sections were incubated for 30 min with HistoVT ONE (Nacalai Tesque, Inc, Kyoto, Japan) for antigen retrieval. After washing with phosphate-buffered saline containing 0.1% Tween 20 (PBT), sections were incubated for 10 min in 3% H_2_O_2_, and for 1 h in in 5% skim milk for blocking. Sections were then incubated for 16 h in anti-monomeric Azami-Green 1 pAb antibody (a polyclonal antibody against GFP of the stony coral *G. fascicularis*; item no. PM052M, Medical & Biological Laboratories, Nagoya, Japan) (1:4,000 in PBT with 2% skim milk) at 4 °C. For the secondary antibody reaction, sections were incubated with a biotinylated goat anti-rat IgG antibody (Vector Laboratories, Burlingame, USA; diluted 1: 2000 in PBT with 2% skim milk) for 30 min. Immunoreactive signals were visualized with avidin–biotin–peroxidase complex (ABC) solution (Vector Laboratories), and 3,30-diaminobenzidine (DAB; Sigma-Aldrich). Sections were counterstained with haematoxylin. Stained sections were observed and photographed under a BX51 microscope (Olympus, Tokyo, Japan).

### Supplementary information

Supplementary information

Description of Additional Supplementary Files

Supplementary Data

reporting-summary

## Data Availability

Raw RNA-sequencing data and raw genomic sequencing data have been deposited in the DDBJ/EMBL/GenBank databases under accession numbers DRR397929–DRR397944 and DRR397945–DRR397946 (BioProject ID: PRJDB14104), respectively. The genome assembly and mitochondrial genome assembly of *F. ancora* have been deposited in the DDBJ/EMBL/GenBank under accession numbers BRZB01000001-BRZB01000205 and LC811399, respectively. Source data of TPM values for the heatmaps shown in Figs. 3, 5, and 6 can be found in the Supplementary Data Sheet 1–5.
