# Supplementary material for: Genome and tissue-specific transcriptomes of the large-polyp coral, Fimbriaphyllia (Euphyllia) ancora: a recipe for a coral polyp
Source: Commun Biol. 2024 Jul 24;7:899. doi: 10.1038/s42003-024-06544-4 (PMC11269664; doi:10.1038/s42003-024-06544-4)
Supplement: Supplementary file 2 — Description of Additional Supplementary Files [file 42003_2024_6544_MOESM2_ESM.pdf]

Description of Additional Supplementary Files

File name: Supplementary Data
Description: Source data of TPM values for the heatmaps shown in Figures 3, 5, and 6.
