# Supplementary material for: Genome and tissue-specific transcriptomes of the large-polyp coral, Fimbriaphyllia (Euphyllia) ancora: a recipe for a coral polyp
Source: Commun Biol. 2024 Jul 24;7:899. doi: 10.1038/s42003-024-06544-4 (PMC11269664; doi:10.1038/s42003-024-06544-4)
Supplement: Supplementary file 4 — reporting-summary [file 42003_2024_6544_MOESM4_ESM.pdf]

nature portfolio

Corresponding author(s): COMMSBIO-23-5014B

Last updated by author(s): Jun 30, 2024

Reporting Summary

Nature Portfolio wishes to improve the reproducibility of the work that we publish. This form provides structure for consistency and transparency in reporting. For further information on Nature Portfolio policies, see our Editorial Policies and the Editorial Policy Checklist.

Statistics

For all statistical analyses, confirm that the following items are present in the figure legend, table legend, main text, or Methods section.

n/a	Confirmed	
☐	☒	The exact sample size (*n*) for each experimental group/condition, given as a discrete number and unit of measurement
☐	☒	A statement on whether measurements were taken from distinct samples or whether the same sample was measured repeatedly
☐	☒	The statistical test(s) used AND whether they are one- or two-sided<br>*Only common tests should be described solely by name; describe more complex techniques in the Methods section.*
☐	☒	A description of all covariates tested
☐	☒	A description of any assumptions or corrections, such as tests of normality and adjustment for multiple comparisons
☐	☒	A full description of the statistical parameters including central tendency (e.g. means) or other basic estimates (e.g. regression coefficient) AND variation (e.g. standard deviation) or associated estimates of uncertainty (e.g. confidence intervals)
☐	☒	For null hypothesis testing, the test statistic (e.g. *F*, *t*, *r*) with confidence intervals, effect sizes, degrees of freedom and *P* value noted<br>*Give P values as exact values whenever suitable.*
☒	☐	For Bayesian analysis, information on the choice of priors and Markov chain Monte Carlo settings
☒	☐	For hierarchical and complex designs, identification of the appropriate level for tests and full reporting of outcomes
☒	☐	Estimates of effect sizes (e.g. Cohen's *d*, Pearson's *r*), indicating how they were calculated

Our web collection on statistics for biologists contains articles on many of the points above.

Software and code

Policy information about availability of computer code

Data collection	n/a
Data analysis	n/a/

For manuscripts utilizing custom algorithms or software that are central to the research but not yet described in published literature, software must be made available to editors and reviewers. We strongly encourage code deposition in a community repository (e.g. GitHub). See the Nature Portfolio guidelines for submitting code & software for further information.

Data

Policy information about availability of data

All manuscripts must include a data availability statement. This statement should provide the following information, where applicable:

- Accession codes, unique identifiers, or web links for publicly available datasets
- A description of any restrictions on data availability
- For clinical datasets or third party data, please ensure that the statement adheres to our policy

Data availability
Raw RNA-sequencing data and raw genomic sequencing data have been deposited in the DDBJ/EMBL/GenBank databases under accession numbers DRR397929–DRR397944 and DRR397945–DRR397946 (BioProject ID: PRJDB14104), respectively. The genome assembly and mitochondrial genome assembly of F. ancora have been deposited in the DDBJ/EMBL/GenBank under accession numbers BRZB01000001-BRZB01000205 and LC811399, respectively. Source data of TPM values for

the heatmaps shown in Figures 3, 5, and 6 can be found in the Supplementary Data Sheet 1-5.

Research involving human participants, their data, or biological material

Policy information about studies with human participants or human data. See also policy information about sex, gender (identity/presentation), and sexual orientation and race, ethnicity and racism.

Reporting on sex and gender	n/a
Reporting on race, ethnicity, or other socially relevant groupings	n/a
Population characteristics	n/a
Recruitment	n/a
Ethics oversight	n/a

Note that full information on the approval of the study protocol must also be provided in the manuscript.

Field-specific reporting

Please select the one below that is the best fit for your research. If you are not sure, read the appropriate sections before making your selection.

☒ Life sciences ☐ Behavioural & social sciences ☐ Ecological, evolutionary & environmental sciences

For a reference copy of the document with all sections, see nature.com/documents/nr-reporting-summary-flat.pdf

Life sciences study design

All studies must disclose on these points even when the disclosure is negative.

Sample size	No sample size calculation was performed. The number of samples (colonies with different genotypes) was set to 4 in order to meet statistical criteria.
Data exclusions	No data were excluded.
Replication	We collected 4 genetically different colonies, and used all of those colony samples for analysis(RNA-seq). One colony represents one biological replicate. So we have four biological replicates.
Randomization	To avoid collecting genetically identical colonies that were produced by asexual reproduction, each colony was sampled at a distance of at least 10 meters from each other. All the collected colonies were used for the analysis.
Blinding	Blinding was not relevant to our study because we used all the collected samples for RNA-seq analysis.

Reporting for specific materials, systems and methods

We require information from authors about some types of materials, experimental systems and methods used in many studies. Here, indicate whether each material, system or method listed is relevant to your study. If you are not sure if a list item applies to your research, read the appropriate section before selecting a response.

Materials & experimental systems

n/a	Involved in the study	
☐	☒	Antibodies
☒	☐	Eukaryotic cell lines
☒	☐	Palaeontology and archaeology
☐	☒	Animals and other organisms
☒	☐	Clinical data
☒	☐	Dual use research of concern
☒	☐	Plants

Methods

n/a	Involved in the study	
☒	☐	ChIP-seq
☒	☐	Flow cytometry
☒	☐	MRI-based neuroimaging

Antibodies

Antibodies used	anti-monomeric Azami-Green 1 pAb antibody (a polyclonal antibody against GFP of the stony coral G. fascicularis; item no. PM052M, Medical & Biological Laboratories, Nagoya, Japan)
Validation	The specific binding of this antibody against GFP was validated by Western blotting and immunohistochemistry using the stony coral, Fimbriaphyllia ancora, samples.

Animals and other research organisms

Policy information about studies involving animals; ARRIVE guidelines recommended for reporting animal research, and Sex and Gender in Research

Laboratory animals	This study did not involve laboratory animals
Wild animals	The stony coral, Fimbriaphyllia ancora, were collected on reef slopes in the nearshore of Onna Village, Okinawa, Japan, under Okinawa prefectural permit #30-8 in 2018. Specimens were collected by divers using a hammer and chisel at depths of 3 m
Reporting on sex	The sex of samples have not been confirmed.
Field-collected samples	The collected coral samples were cultured for 1 day in 100-L aquaria under natural lights. The aquarium was equipped with an external filtration system, and the water temperature was set at 28?.
Ethics oversight	All experiments were carried out in accordance with principles and procedures approved by the Institutional Animal Care and Use Committee, National Taiwan Ocean University.

Note that full information on the approval of the study protocol must also be provided in the manuscript.

Plants

Seed stocks	n/a
Novel plant genotypes	n/a
Authentication	n/a
